# Relationship between the social support and self-efficacy for function ability in patients undergoing primary hip replacement

**DOI:** 10.1186/s13018-018-0857-3

**Published:** 2018-06-18

**Authors:** Kuan-Ting Wu, Pei-Shan Lee, Wen-Yi Chou, Shu-Hua Chen, Yee-Tzu Huang

**Affiliations:** 1 Department of Orthopaedic Surgery, Kaohsiung Chang Gung Memorial Hospital, No.123, Dapi Rd., Niaosong Dist., Kaohsiung city, 833 Taiwan, Republic of China; 2 Department of Orthopedics Operation Room, Kaohsiung Chang Gung Memorial Hospital, No.123, Dapi Rd., Niaosong Dist., Kaohsiung city, 833 Taiwan, Republic of China; 3 Department of Hospital and Health Care Administration, Chia Nan University of Pharmacy and Science, No.60, Sec. 1, Erren Rd., Rende Dist, Tainan city 717, Taiwan, Republic of China

**Keywords:** Self-efficacy for functional ability, Social support, Primary hip replacement, Arthroplasty

## Abstract

**Background:**

The World Health Organization (WHO) reported that nearly 25% of people will suffer from physical disability owing to the bone and joint problems until 2050. The condition of patients with this type of difficulty could be improved by increasing positive self-efficacy and instigating suitable medical treatment to implement self-efficacy for functional ability (SEFA) and physical functional ability self-care. In this study, we aim to evaluate the influence of social support on SEFA in patients after total hip arthroplasty.

**Methods:**

This cross-sectional study used structural questionnaires, telephone appointments, and data collection to obtain patient characteristics, such as gender, age, educational level, and marital status. Questionnaires about social support and self-efficacy for functional ability (SEFA) were sent to 200 patients at 3 months following a primary total hip replacement from September 2011 to December 2014. Factor analysis was used to categorize the dimensions of social support; the *t* test, analysis of variance (ANOVA), and correlation analysis were applied to screen factors influencing SEFA. Multiple regression analysis was employed to ascertain the relationships between patient characteristics, social support, and SEFA.

**Results:**

In total, 134 patients responded to the questionnaires. Lower SEFA scores were observed in patients of an older age, unmarried patients, and those with a low level of education. Correlation analysis showed that emotional information and appraisal support, instrumental support, and SEFA were positively correlated. Multiple regression analysis was applied to ascertain the relationships between patient characteristics, social support, and SEFA. We identified significant coefficient values of − 0.187 for age, 5.344 for emotional information and appraisal support, and 1.653 for instrumental support.

**Conclusion:**

The results of this study demonstrated that in patients undergoing primary hip replacement, positive impacts on SEFA were observed in relation to emotional information, appraisal support and instrumental support. The results indicated that enhancing emotional information and appraisal support could improve a patient’s self-efficacy for functional ability.

## Background

Degenerative hip disease is the most common form of hip discomfort. According to the World Health Organization (WHO), bone and joint diseases may result in physical disability in nearly 25% of the population until 2050. Degenerative osteoarthritis can be diagnosed via physical examination in almost 80% of patients older than 55–65 years, and 20% of these patients already have a disability [1]. Based on research conducted by the Centers for Disease Control and Prevention (CDC) in the USA, the number of patients with osteoarthritis will double in the population over 65 years of age by 2030 [2].

Degenerative osteoarthritis often results in pain and limitation of daily activity, which are the leading causes requiring medical assistance [3]. Initially, these discomforts can be alleviated by appropriate medical management and self-care modification. In addition, caregivers play a key role in psychological support by providing patients with company [4]. In patients who are refractory to conservative treatment, a total hip replacement (THR) is usually indicated. According to the literature, THR yields excellent pain relief, with a survival rate from 90 to 99.8% during a 10–15-year follow-up period [5–7]. However, functional outcome varies according to preoperative characteristics, such as age, comorbidities, and activity level [8]. In addition to surgical technique, postoperative rehabilitation, activity adjustment, and posture limitation are also important for better functional recovery. Psychosocial factors are also associated with recovery after THR [9, 10].

Pain from degenerative osteoarthritis has a negative effect on patients in terms of psychosocial issues in the long term, such as effects on mood and sleep, which interact with activity level [11]. Langford and Bowsher [12] pointed out that adequate social support has positive effects on health status and behavior. In patients with a hip fracture, social support is also correlated with the function of the lower limbs [13]. Self-efficacy represents a patient’s personal confidence in completing specific tasks and also brings about a sense of accomplishment. After lower limb arthroplasty, Moon and Backer identified significant correlations between self-efficacy, walking distance, and lower limb activity frequency [14]. In the elderly population, health status and physical function are key factors affecting the execution of regular exercise; therefore, the condition of patients with this type of difficulty may be improved by increasing positive self-efficacy and social support following THR [15].

In this study, we aimed to examine the relationships between social support, self-efficacy level, and functional ability in patients undergoing primary THR after discharge from the hospital.

## Methods

### Study design and sample

This cross-sectional study used structural questionnaires, telephone appointments, and data collection to obtain patient characteristics, such as gender, age, educational level, and marital status. The Institutional Review Board at our hospital approved this analysis. Patients who had undergone THR due to osteoarthritis of the hip were enrolled from September 2011 to December 2014. Patients with the following conditions were excluded: dementia, psychological disorders, or postoperative complications such as cardiovascular accident, fat embolism, vascular or nerve injury, intraoperative acetabulum, or femur fracture. We used modified versions of the self-efficacy for functional ability (SEFA) scale and the social support scale for patient evaluation.

### Questionnaires

We collected patient characteristics such as gender, age, educational level, and marital status. Self-efficacy for functional ability (SEFA) and social support questionnaires were completed by the patients 3 months following primary THR. As the average age of the patients was greater than 65 years, we used modified versions of the questionnaires for simplification.

### Social support scale

The modified social support scale [16] was revised from the Inventory of Socially Supportive Behaviors (ISSB) developed by Barrera et al. [17] for the measurement of assistance provided in the areas of emotional information, appraisal support, and instrumental support by a spouse, family, friends, and relatives. The patients were asked to respond to 15 items using a Likert scale from 0 to 3 points, representing no assistance, little assistance, much assistance, and very much assistance, respectively. Hence, the global score ranged from 0 to 90, with higher scores indicating better social support. In addition, we categorized social support into two dimensions. Dimension 1 represented emotional information and appraisal support, with a Cronbach’s alpha of 0.973, while dimension 2 represented instrumental support, with a Cronbach’s alpha of 0.767.

### Self-efficacy for functional ability scale

The SEFA scale is usually employed to assess patient confidence in performing daily activities. In this study, we used a modified SEFA proposed by Resnick [18]. Nine items were included in the modified SEFA questionnaire, which assessed functional ability in terms of dressing, getting out of and into bed, using the toilet, bathing, walking, and climbing stairs. Items were scored from 1 to 4, representing patient levels of confidence of less than 20%, 20–50%, 50–80%, and greater than 80%, respectively, in performing the activities by themselves. The total score ranged from 9 to 36, with higher scores representing greater confidence in performing daily activities. The Cronbach’s alpha for this scale was 0.969.

### Statistical analysis

Factor analysis was used to categorize dimensions of social support. The Kaiser-Meyer-Olkin value was 0.898, and the Barlett test showed a *p* value < 0.001. Furthermore, the *t* test, analysis of variance (ANOVA), and correlation analysis were applied to screen the factors influencing SEFA. Multiple regression analysis was employed to ascertain the relationships between patient characteristics, social support, and SEFA. The reliability of the questionnaires was assessed using Cronbach’s alpha; the higher the Cronbach’s alpha value, the greater the internal consistency.

## Results

From September 2011 to December 2014, 200 patients underwent THR for advanced osteoarthritis. Following the exclusion of 66 patients who met the exclusion criteria, 134 patients were enrolled in this study, 51.5% of whom were male; 21.6% of the patients were under 55 years of age, 23.1% were aged between 51 and 64, 35.1% between 65 and 75, and 20.1% were over 75 years of age. 38.1% of the patients had an educational level ranging from elementary school to junior high school, and 26.9% were uneducated. 86.6% of the patients were married (Table 1).

**Table 1:** Characteristics of primary THR patients (*n* = 134)

	Number	Percentage (%)
Sex		
Male	69	51.5
Female	65	48.5
Age (years)		
< 51	29	21.6
51–64	31	23.1
65–75	47	35.1
> 75	27	20.1
Educational level		
Uneducated	36	26.9
Primary/secondary school	51	38.1
> Senior high school	47	35.1
Marital status		
Single	18	13.4
Married	116	86.6

In terms of social support, the average score for the dimension of emotional information and appraisal support was 1.81 ± 0.7, while that for the dimension of instrumental support was 1.56 ± 0.77. The highest score (2.08 ± 0.65) of the items in the dimension of emotional information and appraisal support was obtained for “expressed interest and concern in your well-being,” followed by “reminded you of follow-up hospital appointments” (2.02 ± 0.77). Furthermore, the lowest score was obtained for the item “assisted you in appropriate exercise” (1.37 ± 0.85). In the dimension of instrumental support, the lowest score (1.50 ± 0.75) was obtained for the item “listened to you talk about your private feelings” (Table 2).

**Table 2:** Descriptive analysis of social support after THR

	No assistance (0)	Little assistance (1)	Much assistance (2)	Very much assistance (3)	Mean ± SD
	Number (%)				
Emotional information and appraisal support					1.81 ± 0.70
Expressed interest and concern in your well-being	0 (0%)	23 (17%)	78 (57.8%)	34 (25.2%)	2.08 ± 0.65
Was right there with you in a stressful situation	5 (3.7%)	32 (23.7%)	76 (56.3%)	22 (16.3%)	1.85 ± 0.73
Joked and kidded to try to cheer you up	4 (3%)	23 (17%)	82 (60.7%)	26 (19.3%)	1.96 ± 0.69
Agreed that what you want to do was right	8 (6%)	35 (13.4%)	73 (26.1%)	18 (54.5%)	1.75 ± 0.76
Let you know you did something well	8 (6%)	25 (18.7%)	34 (25.4%)	67 (50%)	1.81 ± 0.80
Expressed esteem or respect for a competency or personal quality of yours	6 (4.5%)	29 (21.6%)	32 (23.9%)	67 (50%)	1.93 ± 0.79
Provided some advice on medical treatment	8 (6%)	30 (22.4%)	31 (23.1%)	65 (48.5%)	1.87 ± 0.82
Provided some advice on daily living	6 (4.5%)	25 (18.7%)	36 (26.9%)	67 (50%)	1.83 ± 0.78
Reminded you of follow-up hospital appointments	6 (4.5%)	20 (14.9%)	35 (26.1%)	73 (54.4%)	2.02 ± 0.77
Assisted you in setting a goal for yourself	13 (9.7%)	26 (19.4%)	47 (35.1%)	48 (35.8%)	1.65 ± 0.90
Did some activity together to help you get your minds off things	24 (17.9%)	26 (19.4%)	27 (20.1%)	57 (42.5%)	1.59 ± 0.99
Helped you in adjustment of daily activities	8 (6%)	32 (23.9%)	42 (31.3%)	52 (38.8%)	1.81 ± 0.87
Assisted you in appropriate exercise	12 (9%)	21 (15.7%)	46 (34.3%)	55 (41%)	1.37 ± 0.58
Instrumental support					1.56 ± 0.77
Listened to you talk about your private feelings	9 (6.7%)	62 (45.9%)	52 (38.5%)	12 (8.9%)	1.50 ± 0.75
Loaned or gave you something that you needed	14 (10.4%)	32 (23.9%)	34 (25.4%)	54 (40.3%)	1.63 ± 0.96

The average SEFA score of the patients after THR was 3.33 ± 0.84. The lowest-scored items in terms of self-efficacy were “wash your lower body (3.24 ± 0.95),” “dress your lower body (3.19 ± 1.00),” and “climb up and down four stairs (2.78 ± 1.21).” The highest score (3.53 ± 0.28) was obtained for “get in and out of bed and chairs” (Table 3). Male patients scored 19.97 ± 8.21 and female patients 21.95 ± 6.84 on average, with a *p* value < 0.01.

**Table 3:** Descriptive analysis of SEFA after THR

Confidence (score)	< 20% (1)	20–50% (2)	50–80% (3)	> 80%(4)	Mean ± SD
Item	Number (%)				
Wash your upper body	8 (6%)	10 (7.5%)	28 (20.9%)	88 (65.7%)	3.46 ± 0.87
Wash your lower body	9 (6.7%)	21 (15.7%)	33 (24.6%)	71 (53%)	3.24 ± 0.95
Dress your upper body	4 (3%)	12 (9%)	26 (19.4%)	92 (68.7%)	3.48 ± 0.92
Dress your lower body	9 (6.7%)	24 (17.9%)	29 (21.6%)	72 (53.7%)	3.19 ± 1.00
Get on and off the toilet and manage your clothes	8 (6%)	13 (9.7%)	20 (14.9%)	93 (69.4%)	3.48 ± 0.90
Get in and out of bed and a chair	4 (3%)	8 (6%)	31 (23.1%)	91 (67.9%)	3.53 ± 0.82
Walk 50 ft	4 (3%)	12 (9%)	32 (23.9%)	86 (64.2%)	3.43 ± 0.92
Walk 120 ft	6 (4.5%)	12 (9%)	37 (27.6%)	79 (59%)	3.37 ± 0.93
Climb up and down four stairs	22 (16.4%)	26 (19.4%)	31 (23.1%)	55 (41%)	2.78 ± 1.21
Total score					29.96 ± 7.6
Total average score					3.33 ± 0.84

A positive correlation between emotional information and appraisal support with instrumental support was observed, with a Pearson correlation coefficient of 0.6. In addition, a positive relationship between emotional information and appraisal support with SEFA was seen, with a Pearson correlation coefficient of 0.635 (Table 4).

**Table 4:** Pearson’s correlations with SEFA scale

Variable	Correlation coefficient, *r*	*p* value
Emotional information and appraisal support	0.635	< 0.001
Instrumental support	0.489	< 0.001

Scores on the scales of self-efficacy (17.58 ± 8.98) and emotional information and appraisal support (1.46 ± 0.80) were lower in the uneducated patients (*p* < 0.01), as was the instrumental support score (*p* < 0.05). In addition, the elderly patients had lower SEFA scores (*p* < 0.01) (Table 5).

**Table 5:** Group comparison of emotional information and appraisal support, instrumental support and SEFA

Variable	Emotional information and appraisal support		Instrumental support		Self-efficacy of functional ability	
	M ± SD	*t*, *F* value	M ± SD	*t*, *F* value	M ± SD	*t*, *F* value
Sex (T)		4.454		0.563**		3.487**
Male	1.66 ± 0.75		1.35 ± 0.73		19.91 ± 8.21	
Female	1.97 ± 0.61		1.80 ± 0.75		21.95 ± 6.84	
Age (years)(A)		0.954		0.197		8.123**
(1) < 50	1.88 ± 0.74		1.64 ± 0.91		23.83 ± 4.65	
(2) 51–64	1.74 ± 0.56		1.48 ± 0.46		21.27 ± 9.18	
(3) 65–75	1.90 ± 0.68		1.55 ± 0.79		22.10 ± 4.63	
(4) > 75	1.64 ± 0.83		1.58 ± 0.91		15.04 ± 9.74	
Post hoc Scheff					(4) < (1), (2), (3)	
Educational level (A)		8.978**		4.430*		7.476*
(1) Uneducated	1.46 ± 0.80		1.39 ± 0.73		17.58 ± 9.00	
(2) Primary to secondary school	2.07 ± 0.50		1.81 ± 0.73		23.65 ± 4.97	
(3) > Senior high school	1.79 ± 0.72		1.43 ± 0.80		20.12 ± 8.16	
Post hoc Scheff	(1) < (2)		(1) < (2)		(1) < (2)	
Marital status (T)		3.502		0.284		8.182*
Single	1.88 ± 0.46		1.64 ± 0.68		20.51 ± 7.90	
Married	1.80 ± 0.73		1.55 ± 0.79		23.83 ± 4.40	

A represents ANOVA and T represents independent *t* test

*p < 0.05*, **p < 0.01

We included predictors selected from *t* tests and ANOVA, such as sex, age, educational level, marital status, emotional information, appraisal support, and instrumental support in stepwise multivariable regression analysis. After the elimination procedure, age, educational level, emotional information and appraisal support, and instrumental support were selected as significant predictors. These significant predictors were analyzed using an all-possible regression procedure. It was found that an older age was related to a lower self-efficacy (*b* = − 0.187, *p* < 0.001). Emotional information and appraisal support had a positive influence on self-efficacy (*b* = 5.344, *p* < 0.001), as did instrumental support (*b* = 1.653, *p* < 0.05). The model explained approximately 52.6% of the variance in SEFA 3 months after THR, and the final regression model explained 50.7% of the variance (Table 6).

**Table 6:** Multivariable regression analysis of SEFA

SEFA			
Variable	B	S(B)	VIF
Patient characteristics			
Age	− 0.187***	− 0.333	1.355
Educational level			
Uneducated (reference)			
Primary/secondary school	− 0.039	− 0.003	2.038
> Senior high school	− 2.644	− 0.161	1.875
Social support			
Emotional information and appraisal support	5.344***	0.491	1.714
Instrumental support	1.653*	0.166	1.609

*R*^2^ = 0.526, adjust *R*^2^ = 0.507, *n* = 134

*p < 0.05, **p < 0.01, *** *p* < 0.0001

## Discussion

Social support is a key factor related to the daily activity of geriatric patients. Friedland and McColl [19] pointed out that interventions to increase social support in critical patients can improve chronic stress and physical disability. Social support also has positive effects on health status and behavior [12] and can enhance execution of healthy behaviors, such as taking medicines on time, engaging in regular exercise, and diet control [20]. In studies of patients with hip fractures, a significant relationship was also identified between social support and lower limb functional activity [13], especially when supported by their spouse and family [21]. Social support is usually provided by the patient’s family members who live with them. Kiefer identified a higher level of social support in patients living with a spouse, adult child, or friends while the singles had a lower level of social support [22]. In this study, we found that the item with the highest score (2.08 ± 0.65) was “expressed interest and concern in your well-being.” Because it is easier for a caregiver or family to focus on the patient’s disease-related health status, as they can directly observe their gait, muscle power, and agility following THR. Sveikata et al. [23] demonstrated better postoperative functional results 12 months after total knee arthroplasty in patients who had better social support. McHugh et al. [24] also identified key psychosocial factors and biomedical predictors of pain, anxiety, and depression related to recovery following THR in 206 patients. Social support can affect self-efficacy in terms of verbal encouragement. In a randomized control study, improved general self-efficacy and physical function were observed in the group who received telephone follow-up appointments conducted by a nurse, which was structured along the lines of the VIPS model (the Swedish acronym for the concepts of Well-being, Integrity, Prevention and Safety) [25]. We also identified a significant positive correlation between social support and self-efficacy in our study, with a coefficient of 5.344 for emotional information and appraisal support and 1.653 for instrumental support.

Physical activity is crucial in the elderly and can prevent chronic disease, promote physical health, and help to maintain the quality of life [26]. Elderly persons who engage in regular exercise will perform better in functions of daily activity and will have an improved health status and reduce few chronic diseases such as stroke, cardiovascular disease, osteoporosis, hypertension, and diabetes [27, 28]. If patients are restricted in terms of the execution of physical activity, they will be unwilling to perform daily activities [29]. Similar with social support, increase self-efficacy is associated with higher physical activity levels [30, 31]. Poor self-efficacy usually leads to greater distress in patients during their recovery; they often worry about their condition getting worse after executing a specific task because they are not confident in their course of rehabilitation. In research into self-efficacy, self-efficacy was taken as an evaluation of capability in terms of achievement in specific tasks, a significant correlation between self-efficacy and personal success was identified [32, 33]. Dominick et al. demonstrated a significant correlation between low self-efficacy for exercise and less improvement in functional recovery over time after total knee arthroplasty [34]. Waldrop et al. [35] also addressed self-efficacy predicted significant variance in rehabilitation outcomes in orthopedic surgery. They concluded that augmenting self-efficacy beliefs by psychologists could improve functional recovery. The higher the level of induced self-efficacy, the greater the performance accomplishments.

Patients who undergo THR are usually discharged home directly. Therefore, it is easy to assess the correlation of functional outcome with the quality of home care. Self-efficacy was identified as a significant predictor of recovery after joint arthroplasty by Moon and Backer et al. [14]. They also found that walking distance and the frequency of lower limb activity increased as self-efficacy improved. In our study, the SEFA item with the highest score was “get in and out of bed and chairs,” while the lowest-scored item was “climb up and down four stairs”. As climbing stairs requires high levels of coordination and strength, it is difficult for patients to complete this task with ease. Self-efficacy might be affected in several ways. According to the literature, there exist two factors that influence the daily activity and self-efficacy of patients who have undergone THR: patient characteristics and disease characteristics. In terms of patient characteristics, confidence in self-efficacy decreases with aging [36, 37]. Janiszewska et al. demonstrated that older age deteriorated general health and decreased general self-efficacy level in women who undergone osteoporosis treatment [38]. With regard to the level of education, Lien and Wei studied 350 patients separated into diabetic and non-diabetic groups, and a better self-efficacy was associated with a higher educational level [39]. Marital status also affects self-efficacy in correlation with social support. Fitzgerald et al. [40] evaluated 222 patients 12 months after joint replacement surgery, and better social support and self-efficacy were noted in patients who were married or living with someone. Regarding disease characteristics, patients have difficulties in performing daily activities owing to specific posture limitations during the first 6 to 12 weeks following THR [41, 42]. In our study, we evaluated patients 3 months following THR in order to minimize bias and observed better SEFA in married patients and those with a higher level of education.

Some studies have identified different contributions of preoperative and postoperative self-efficacy to patient outcome following THR [43–45]. A higher preoperative self-efficacy means that the patient has more confidence in their recovery and is willing to undergo THR; furthermore, preoperative self-efficacy has also been identified as a predictor of better recovery. However, in a systemic review regarding the influence of self-efficacy on functional recovery, the effect of preoperative self-efficacy was inconclusive, while in contrast, the effect of postoperative self-efficacy was consistent and was found to be associated with functional recovery in terms of distance ambulation, exercise repetition, and frequency [45, 46]. Some studies have indicated that self-efficacy is associated with emotional outcomes [47]. In our study, we attempted to identify factors correlated with postoperative SEFA; therefore, we evaluated the relationships of emotional information and instrumental support with postoperative SEFA and identified significant correlations, with Pearson coefficients of 0.635 and 0.483, respectively.

### Limitations

First, we used modified questionnaires in order to make it easier for the elderly participants to respond to items. The validity of the SEFA and social support questionnaires was examined, and both had a high internal consistency, with Cronbach’s alpha values greater than 0.7; however, these values were still lower than those of the primary versions of the questionnaires. Second, all patients were enrolled from a single hospital, and differences in culture in other cities might result in differences in social support and self-efficacy.

## Conclusion

This study concluded that in patients undergoing primary hip replacement, emotional information, appraisal support, and instrumental support had positive impacts on self-efficacy for functional ability. The results indicated that enhancing emotional information and appraisal support could improve self-efficacy for functional ability and lead to a better recovery following THR.

## Abbreviations

CDC: Centers for Disease Control and Prevention
SEFA: Self-efficacy for functional ability
THR: Total hip replacement
WHO: World Health Organization
